# Staphylococcus aureus Pyogenic Spondylodiscitis in a Patient With Rheumatoid Arthritis Receiving Rituximab

**DOI:** 10.7759/cureus.108803

**Published:** 2026-05-13

**Authors:** El Marbouh El Kacem, Abderrahim Majjad, Meryem Edderai, Hamza Toufik, Laila Taoubane, Ahmed Bezza

**Affiliations:** 1 Rheumatology, Mohammed V Military Training Hospital, Rabat, MAR; 2 Radiology, Mohammed V Military Training Hospital, Rabat, MAR

**Keywords:** immunosuppression, rheumatoid arthritis, rituximab, spondylodiscitis, staphylococcus aureus

## Abstract

We report the case of a 55-year-old woman with seropositive rheumatoid arthritis (RA) treated with methotrexate and rituximab (RTX) who developed severe inflammatory low back pain one month after her fourth RTX cycle. MRI of the lumbosacral spine revealed L4-L5 infectious spondylodiscitis with vertebral endplate destruction, anterior epidural extension, and a right psoas abscess. CT-guided aspiration confirmed methicillin-sensitive *Staphylococcus aureus* infection. Empirical IV therapy with ciprofloxacin and gentamicin was initiated per local protocol. Following microbiological confirmation, ciprofloxacin was continued as the targeted regimen based on confirmed susceptibility (minimum inhibitory concentration (MIC) ≤ 1 mg/L), and gentamicin was discontinued after five days. Both RTX and methotrexate were temporarily suspended. After four weeks of IV therapy, inflammatory markers normalized, and the patient was discharged to complete a three-month antibiotic course, with a favorable outcome. This case highlights the importance of considering pyogenic spondylodiscitis in RTX-treated patients presenting with acute spinal pain, even in the absence of overt hypogammaglobulinemia.

## Introduction

Infectious spondylodiscitis is a severe spinal infection associated with significant morbidity and mortality, with an incidence of approximately three cases per 100,000 inhabitants per year, a rate that appears to be rising, particularly among immunocompromised and multimorbid patients [1]. *Staphylococcus aureus* is the most frequently isolated pathogen in pyogenic vertebral osteomyelitis, accounting for up to 64% of pyogenic cases in recent series, and is associated with a high risk of treatment failure, especially in the presence of local abscess formation [1,2]. In immunocompromised patients, including those receiving biologic therapies, corticosteroids, or cytotoxic agents, pyogenic spinal infections may present atypically and with significant diagnostic delay and should be considered early, even in the absence of classic features such as fever [3]. Rituximab (RTX) is a chimeric anti-CD20 monoclonal antibody widely used in the treatment of hematologic malignancies and autoimmune diseases, particularly rheumatoid arthritis (RA). Its mechanism of action is based on rapid and sustained B-cell depletion, typically persisting for 6 to 12 months, with B-cell recovery generally occurring after 12 months [4]. While RTX is associated with an increased risk of serious infections, estimated at approximately 5.0 per 100 patient-years in RA patients according to the AutoImmunity and Rituximab (AIR) registry [5], this risk is predominantly described in the context of respiratory and opportunistic infections. By contrast, pyogenic spinal infections in RTX-treated RA patients are rarely reported in the literature and may be underrecognized due to their insidious onset and the potential masking of inflammatory signs by ongoing immunosuppression. A focused PubMed search using the terms “spondylodiscitis,” “vertebral osteomyelitis,” “rituximab,” and “rheumatoid arthritis” did not identify any previously published case specifically describing this association, further underscoring the rarity and potential underreporting of this complication.

We report a case of *S. aureus* pyogenic spondylodiscitis in a patient receiving RTX therapy for RA, aiming to raise clinician awareness of this potentially severe and underreported infectious complication.

## Case presentation

A 55-year-old woman with a long-standing history of seropositive RA and hypertension presented with severe inflammatory low back pain (visual analog scale (VAS) 9/10). Her RA was managed with methotrexate (25 mg/week) and RTX (1 g on D0 and D15 every 12 months). The pain was exacerbated by movement; however, she denied any limb weakness, saddle anesthesia, or bowel or bladder dysfunction. The patient had a history of mechanical low back pain since 2002. One month after her fourth RTX cycle, the pain became inflammatory and markedly worsened.

Her RA had been evolving since 2012, with a Disease Activity Score in 28 joints using C-reactive protein (DAS28-CRP) of 4.6 prior to the infectious episode, indicating high disease activity. RTX was her first biologic therapy, initiated after an inadequate response to methotrexate alone; no prior biologic disease-modifying antirheumatic drug (bDMARD) exposure was documented. Long-term corticosteroids had been used prior to 2018 but were discontinued following the introduction of RTX. No corticosteroids or nonsteroidal anti-inflammatory drugs (NSAIDs) were being administered at the time of infection. Pneumococcal and influenza vaccinations had been performed in 2018 prior to RTX initiation, in accordance with current recommendations; however, booster doses had not been administered at the five-year interval. A thorough clinical evaluation was performed to identify a potential portal of entry for *S. aureus*, including assessment for diabetes, renal disease, skin infection, dental infection, urinary tract infection, recent invasive procedures, vascular catheters, and trauma. No alternative source of bacteremia was identified, supporting a cryptogenic hematogenous origin.

Upon admission, the patient was afebrile (37°C) and hemodynamically stable, with a heart rate of 80 bpm, blood pressure of 130/72 mmHg, and respiratory rate of 18 breaths per minute. Physical examination showed diffuse lumbar tenderness, more pronounced in the lower segments.

Initial laboratory tests showed leukocytosis, with a white blood cell count of 15,800/mm³, and neutrophil predominance at 67%. Inflammatory markers were markedly elevated, with a CRP level of 103 mg/L. Immunological workup revealed that immunoglobulin G, A, and M levels were all within normal limits, with no evidence of hypogammaglobulinemia (Table 1).

**Table 1:** Initial laboratory results.

Parameter	Patient's value	Reference range
WBC count	15,800/mm³	4,000-10,000/mm³
Neutrophils	67%	50%-70%
CRP	103 mg/L	<5 mg/L
Immunoglobulin G (IgG)	9.56 g/L	7.0-16.0 g/L
Immunoglobulin A (IgA)	3.22 g/L	0.7-4.0 g/L
Immunoglobulin M (IgM)	0.35 g/L	0.33-2.3 g/L

**Figure 1:**
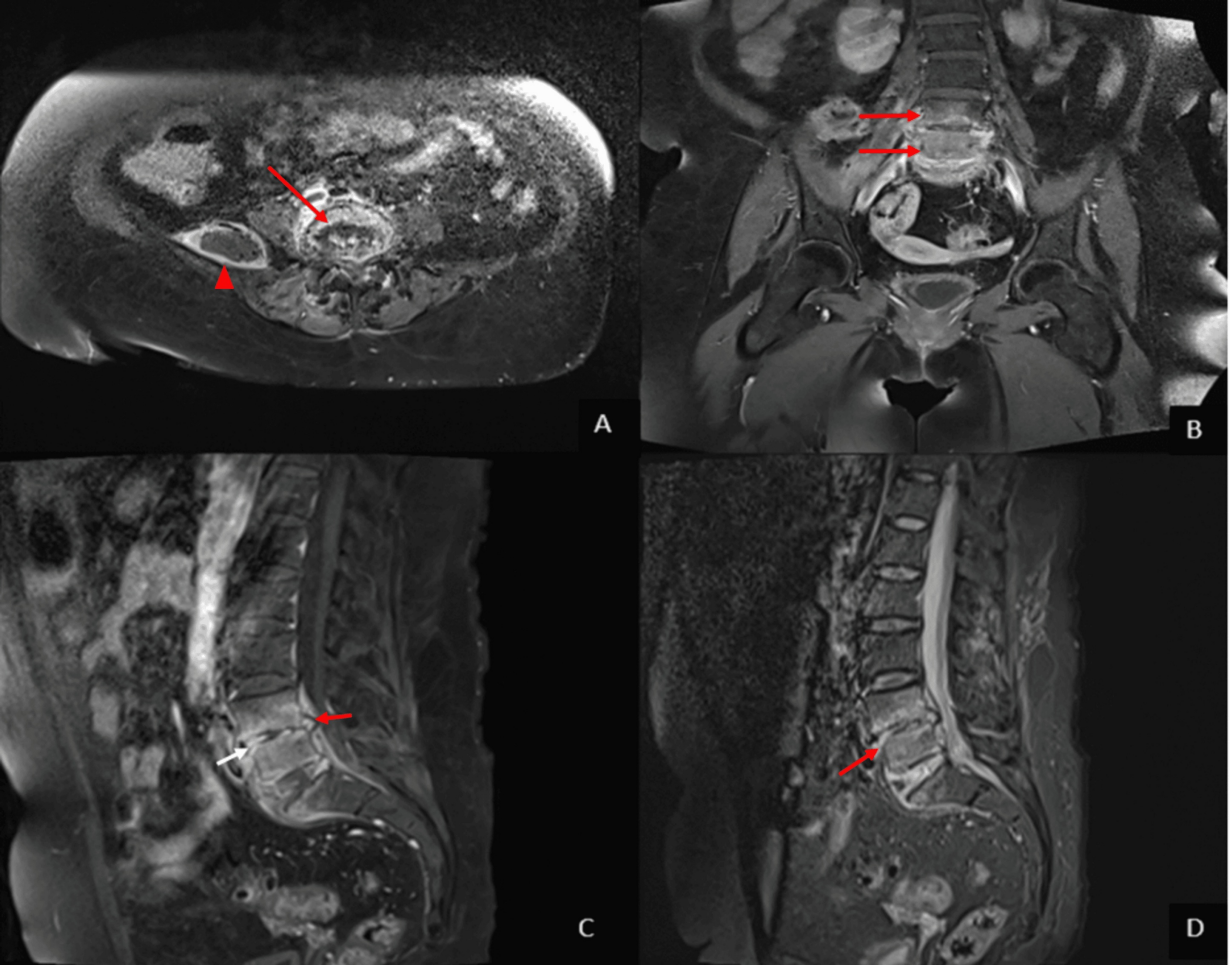
Multimodal MRI findings of L4-L5 infectious spondylodiscitis. (A) Axial T2-weighted fat-saturated image: High-signal intensity in the disc space (red arrow) and a well-defined fluid collection in the right paravertebral space (arrowhead), confirming an organized abscess rather than a diffuse phlegmon. (B) Coronal T2-weighted STIR image: Extensive bone marrow edema in the L4 and L5 vertebral bodies (red arrows), with inflammatory infiltration extending toward the right psoas muscle. (C) Post-contrast sagittal T1-weighted fat-saturated image: Anterior epidural involvement (epiduritis) is visible (red arrow) in continuity with the infected L4-L5 disc space (white arrow). Despite this extension, there was no significant spinal canal stenosis or neural compression, correlating with the lack of neurological deficits. (D) Sagittal T2-weighted fat-saturated image: Diffuse hyperintensity within the L4-L5 disc and adjacent vertebrae (red arrow), associated with grade I anterolisthesis of L4 on L5. STIR: Short tau inversion recovery.

MRI of the lumbosacral spine showed findings consistent with L4-L5 infectious spondylodiscitis, characterized by vertebral endplate erosions; anterior epidural extension without significant canal stenosis or neural compression, consistent with the patient’s intact neurological examination; and a well-defined organized paravertebral abscess with involvement of the adjacent psoas muscle, occurring alongside grade I anterolisthesis at the same level (Figure 1).

Regarding the management of her autoimmune condition, both RTX and methotrexate were immediately suspended to facilitate immune recovery during the infection. No RA flare was observed during hospitalization, allowing for focused treatment of the spondylodiscitis. Upon completion of the three-month antibiotic course, once full clinical and biological resolution was confirmed, methotrexate alone was restarted as maintenance therapy. RTX was not resumed. Disease activity at the time of methotrexate resumption was assessed at a DAS28-CRP of 2.2, indicating remission. No alternative biologic therapy was required during the treatment period.

**Figure 2:**
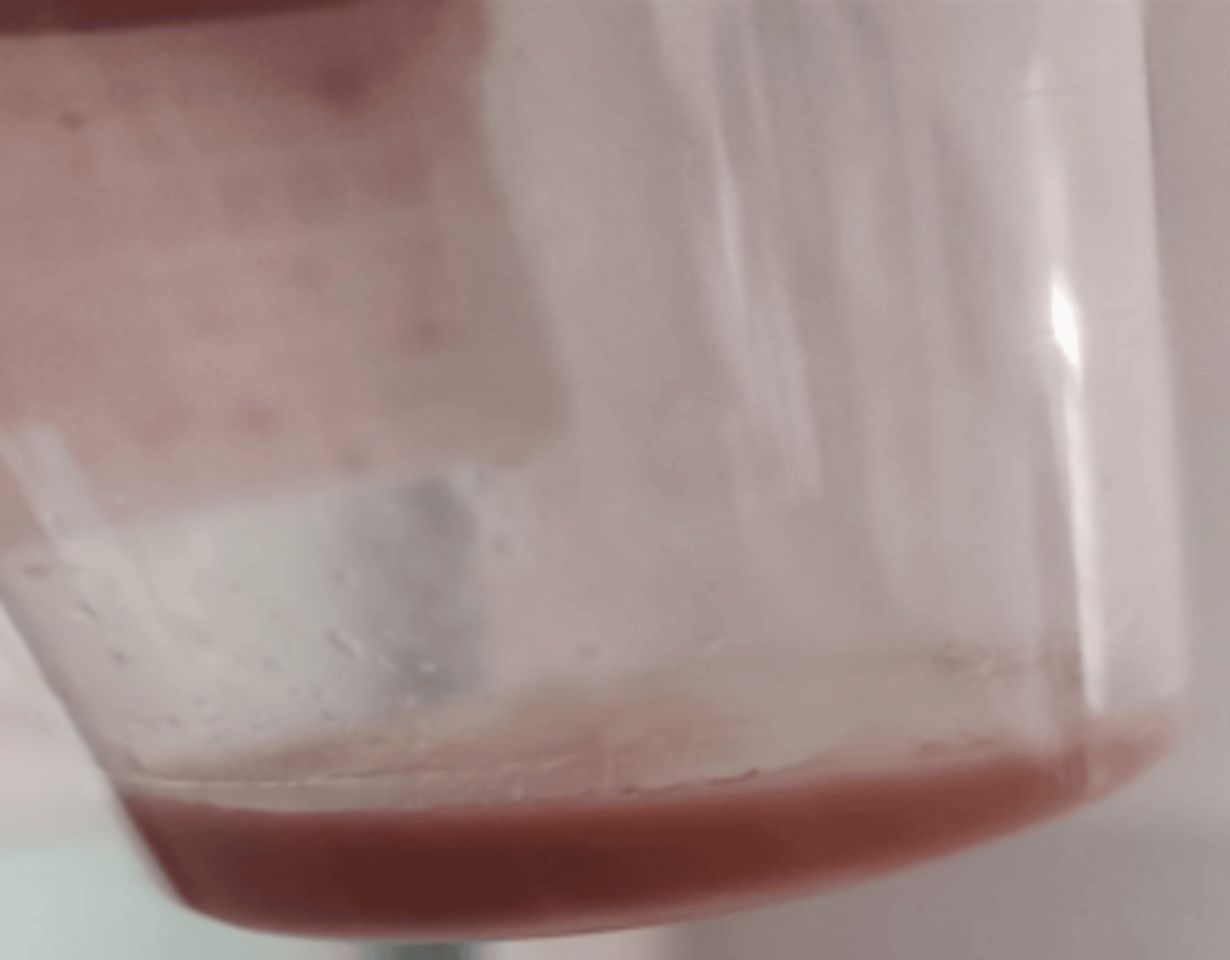
Macroscopic appearance of the psoas abscess aspirate. CT-guided needle aspiration yielded a thick, reddish-brown purulent fluid from the right psoas collection. The turbid and hemorrhagic appearance is highly suggestive of an active pyogenic infection.

A CT-guided needle aspiration of the right psoas abscess was performed under local anesthesia via a posterolateral approach, yielding purulent fluid (Figure 2), with subsequent cultures isolating *S. aureus*.

Pending microbiological identification and susceptibility results, empirical IV antibiotic therapy was initiated according to the local protocol for suspected hematogenous vertebral osteomyelitis. The regimen consisted of ciprofloxacin (800 mg/day) and gentamicin (160 mg/day), providing broad-spectrum coverage against Gram-negative organisms while awaiting definitive culture results. Following microbiological confirmation of methicillin-sensitive *S. aureus* with confirmed susceptibility to ciprofloxacin (minimum inhibitory concentration (MIC) ≤ 1 mg/L), the empirical regimen was maintained as targeted therapy. Anti-staphylococcal beta-lactams (oxacillin and cloxacillin) were not available on the local susceptibility testing panel at the time of management. Management decisions were discussed with the local infectious diseases team and adapted to institutional antimicrobial availability and susceptibility testing practices. Gentamicin was discontinued after five days; serum creatinine and eGFR were monitored throughout and remained within normal limits, with no evidence of nephrotoxicity.

Given the absence of neurological deficits and the non-compressive nature of the epidural extension, conservative management was pursued without indication for formal surgical management. The patient was immobilized with a dorsolumbar brace and received tramadol analgesia throughout the three-week hospitalization. Rehabilitation was initiated from admission with lower limb physiotherapy, and progressive verticalization was commenced after seven days of antibiotic therapy.

After four weeks of IV therapy, inflammatory markers normalized, with a CRP level of 4 mg/L. The patient was then discharged on oral ciprofloxacin (1 g/day) to complete a total of three months of treatment. The clinical and biological outcome was favorable.

A structured clinical timeline summarizing all key events from RTX administration through the end of antibiotic therapy and follow-up is provided in Table 2.

**Table 2:** Clinical timeline. VAS: Visual analog scale; MSSA: Methicillin-sensitive *Staphylococcus aureus*; MIC: Minimum inhibitory concentration; DAS28-CRP: Disease Activity Score in 28 joints using C-reactive protein.

Date	Event
October 2025	Fourth rituximab cycle administered
November 2025	Onset of severe inflammatory low back pain (VAS 9/10)
December 8, 2025	Hospital admission
December 11, 2025	MRI of the lumbosacral spine: L4-L5 infectious spondylodiscitis with epidural extension and right psoas abscess
December 15, 2025	CT-guided aspiration of the psoas abscess; empirical IV ciprofloxacin (800 mg/day) plus gentamicin (160 mg/day) initiated
December 17, 2025	Culture result: MSSA confirmed; susceptibility to ciprofloxacin confirmed (MIC ≤ 1 mg/L); regimen maintained
December 20, 2025	Gentamicin discontinued after five days; renal function remained normal throughout
January 19, 2026	CRP normalization (4 mg/L), five weeks after aspiration
January 26, 2026	Discharge on oral ciprofloxacin (1 g/day), at the end of the sixth week post-aspiration
February 9, 2026	Follow-up visit, 14 days post-discharge: CRP 4 mg/L; favorable outcome
March 9, 2026	Follow-up visit, one month after the previous visit: CRP 4.5 mg/L; continued favorable outcome
March 15, 2026	End of antibiotic therapy; three-month course completed; methotrexate restarted; DAS28-CRP 2.2

## Discussion

The increasing use of bDMARDs has improved RA outcomes but has also increased the risk of infections. Among these, rituximab (RTX), a chimeric monoclonal antibody targeting CD20-positive B cells, is associated with a specific risk profile, primarily involving pyogenic and viral infections due to prolonged B-cell depletion [6].

RTX induces profound and sustained depletion of peripheral B lymphocytes, typically lasting six to 12 months. While it is highly effective in managing refractory RA, it impairs the humoral immune response. According to the Autoimmunity and Rituximab (AIR) registry, serious infections occur at a rate of approximately 5.0 per 100 patient-years in RA patients treated with RTX [5].

*S. aureus* remains the most frequently isolated pathogen in pyogenic spondylodiscitis, accounting for up to 50% of cases in some series, and its occurrence is significantly higher in immunocompromised hosts [1].

B-cell depletion induced by RTX alters B-cell homeostasis and the B-cell activating factor (BAFF)-mediated cytokine environment, leading to impaired B-cell maturation and delayed memory B-cell reconstitution. Although long-lived plasma cells are spared and total immunoglobulin levels may remain within normal ranges, disruption of humoral immunity may increase susceptibility to infections, particularly pyogenic bacterial infections [6,7]. Clinicians should remain vigilant for infection regardless of a reassuring immunological workup.

The diagnosis of spondylodiscitis is often delayed, with a mean diagnostic lag of two to six months. In our patient, the rapid progression of symptoms within one month of RTX infusion may suggest a more rapid disease course in immunosuppressed patients. MRI remains the gold standard for diagnosis, with sensitivity and specificity exceeding 90% [8].

Several limitations of this report merit consideration. First, while the temporal relationship between RTX administration and the onset of spondylodiscitis is suggestive, a definitive causal link cannot be established from a single case. The patient had previously received long-term corticosteroid therapy, which was discontinued in 2018 following the introduction of RTX cycles. Although corticosteroids were no longer administered at the time of infection, their prior prolonged use may have contributed to cumulative immune dysregulation, making it difficult to attribute the infectious complication solely to RTX. Other potential contributing factors, including an unidentified portal of entry for *S. aureus* bacteremia, could not be fully excluded despite a thorough clinical evaluation. Second, the diagnosis of infectious spondylodiscitis in patients with RA carries inherent diagnostic uncertainty, as inflammatory spinal involvement may mimic rheumatoid axial disease, potentially delaying recognition and microbiological confirmation. Third, the absence of hypogammaglobulinemia in our patient challenges the assumption that humoral immune deficiency is a prerequisite for serious infection in RTX-treated patients and suggests that functional B-cell impairment may be sufficient to increase infectious susceptibility even with preserved immunoglobulin levels. These considerations highlight the importance of cautious causal inference when reporting single cases and underscore the need for larger registry-based studies to better characterize the true incidence and risk factors of pyogenic spinal infections in RTX-treated patients.

The management of spondylodiscitis in RA requires a dual approach: targeted antimicrobial therapy and the temporary suspension of immunosuppressive agents. Current guidelines suggest that bDMARDs should be withheld during an active serious infection until clinical and biological resolution is achieved [9]. In our case, the prompt discontinuation of RTX, combined with a three-month course of targeted antibiotic therapy based on susceptibility testing, with ciprofloxacin used according to local protocol, led to a favorable outcome without triggering an RA flare. This suggests that the “drug hold” strategy is safe and effective for managing serious infectious complications in stable RA patients.

## Conclusions

This case shows that pyogenic spondylodiscitis can occur in patients receiving RTX for RA, even in the absence of overt hypogammaglobulinemia. It highlights the importance of considering serious bacterial infections in immunomodulated patients presenting with acute spinal pain. Early recognition, prompt microbiological confirmation, and appropriate antimicrobial therapy, together with the temporary interruption of immunosuppressive agents, were associated with a favorable clinical outcome in this case.
